# Persistence of related bla-IMP-4 metallo-beta-lactamase producing Enterobacteriaceae from clinical and environmental specimens within a burns unit in Australia - a six-year retrospective study

**DOI:** 10.1186/2047-2994-2-35

**Published:** 2013-12-18

**Authors:** Grace HY Leung, Timothy J Gray, Elaine YL Cheong, Peter Haertsch, Thomas Gottlieb

**Affiliations:** 1 Department of Microbiology and Infectious Diseases, Sydney Local Health District Concord Hospital Human Research Ethics Committee, Hospital Rd, Concord, Sydney, NSW 2139, Australia; 2 NSW Severe Burn Injury Service, Concord Hospital, Sydney, Australia

**Keywords:** Carbapenem-resistant Enterobacteriaceae, *bla*-IMP-4 metallo-beta-lactamase enzyme, Hospital-acquired infection, Infection control, Environmental surveillance

## Abstract

**Background:**

To describe the clinical epidemiology, environmental surveillance and infection control interventions undertaken in a six-year persistence of *bla*-IMP-4 metallo-beta-lactamase (MBL) producing Enterobacteriaceae within a separately confined hospital burns unit in a tertiary hospital in Sydney, Australia.

**Methods:**

MBL positive clinical and environmental isolates were collected from the Burns Unit, from the first detection of isolates in September 2006 to August 2012. Unit-acquired clinical isolates were included, and patient outcomes analyzed amongst those who acquired clinically significant infections. Environmental isolates were analyzed with regard to relationship to clinical isolates, bacterial species, and persistence despite cleaning efforts.

**Results:**

Thirty clinical isolates detected from 23 patients were identified. Clinically significant infection developed in 7 (30%) patients – 2 bacteremias, 2 central venous catheter tip infections without bacteremia, and 3 wound infections. All patients survived at 30 days. Seventy-one environmental isolates were confirmed to be MBL-positive, with 85% sourced from shower facilities or equipment. MBL organisms persisted at these sites despite both usual hospital cleaning, and following targeted environmental disinfection interventions.

**Conclusions:**

Clear association exists between environmental Burns Unit contamination by MBLs and subsequent patient colonization. Clinical infection occurred in a small proportion of patients colonized by MBLs, and with generally favorable outcomes. Its persistence in the Burns Unit environment, despite concerted infection control measures, pose concern for ongoing clinical transmission.

## Background

Carbapenem-resistant Enterobacteriaceae (CRE) have emerged as a global issue, particularly in the nosocomial setting [1]. *Bla*-IMP-4 type Ambler Class B metallo-beta-lactamases (MBL) are the predominant type detected in Australia [2,3], after being first identified in 2002 [4]. Apart from a well-described clinical outbreak in 2004 [5,6], and a recent description of an outbreak in an intensive care unit potentially attributed to hand-washing sinks [7], there have been few publications describing MBL outbreaks in the Australian clinical setting. Worldwide there have been other published outbreak examples, due to multi-drug resistant Gram-negative bacteria including *Pseudomonas aeruginosa*, extended-spectrum beta-lactamases (ESBLs) and carbapenemases [8-10].

A recent study by Betteridge et al. [11] performed on specimens from the Concord Repatriation General Hospital (CRGH) Burns Unit compared clinical and environmental MBL isolates and confirmed the strains to be genetically identical, confirming the hospital environment as an important reservoir. This study describes the clinical outbreak, the environmental surveillance performed, and the infection control interventions undertaken.

## Methods

### Setting

CRGH is a 550-bed tertiary hospital in Sydney Australia, and contains the statewide referral unit for severe burns injury, which receives 200 to 300 admissions per year. The Burns Unit (BU) is an enclosed unit containing 8 single rooms and 3 two-bedded rooms. The single rooms are attached to a separate anteroom for hand-washing and donning standard contact precautions (gowns and gloves), which are mandatory regardless of multi-resistant organism (MRO) colonization status. The two-bedded rooms are infrequently used. Five shower facilities are shared amongst the patients. A dedicated burns operating theatre is located adjacent to the Unit. Patients with severe burns are frequently admitted into the intensive care unit (ICU) prior to transfer to BU; inter-hospital transfers represent approximately 50% of presentations.

Routine screening of patients for MBL organisms was not performed during the study period; patient specimens were collected based on clinical need. Environmental screening for Gram negative MROs within patient rooms occurred following patient discharge from the BU and terminal room disinfection. Other specimens were collected from sites within shared shower rooms and sluices. Regular itemized point-prevalence environmental screening was adopted within the BU from October 2011.

In September 2006, a patient known to be colonized with *bla*-IMP-4 MBL *Enterobacter cloacae* was transferred from another hospital to the BU. The IMP-4 positive status was confirmed on wound swabs performed on arrival, and became the index MBL-positive case for the hospital. Since this time, there has been ongoing MBL acquisition among patients admitted into the BU. We describe the clinical epidemiology of this outbreak since this time, until the end of our study period in August 2012.

### Study inclusion

All MBL isolates detected in the hospital within the study period were identified. “Burns-related” isolates were included, and defined as isolates from patients who had dwelled in the BU prior to, or at the time of collection during that same admission. Of these, only original isolates were included. An original isolate was defined as 1) the first MBL isolate detected in that patient, 2) subsequent MBL isolate from a patient, but detected in a new site, e.g. blood cultures from a patient known to be MBL from the skin; this was included to detect possible clinical infection after colonization, and/or 3) subsequent MBL isolates from a patient, but detected in new genus, e.g. MBL-producing *Klebsiella pneumoniae* in a patient previously colonized with MBL-producing *Enterobacter cloacae*. From the included isolates, patient records were identified to determine clinical significance, treatment and outcomes.

Exclusion criteria included 1) patients who were found to harbor an MBL isolate on arrival to the BU, suggesting prior acquisition from another source; and 2) subsequent isolates from the same site, such as repeat skin swabs from a patient known to have MBL-producing organisms colonized on the skin, and 3) carbapenem-resistant organisms such as *Acinetobacter*, *Stenotrophomonas* and *Pseudomonas* spp., in which MBL enzyme presence was excluded on laboratory testing.

Environmental isolates obtained from the BU during this study period retrospectively analysed, and included if laboratory testing had confirmed the presence of MBL enzyme.

### Laboratory methods

Clinical specimens, including wound cultures, underwent routine culture using Horse Blood and MacConkey agars. Highly selective differential and chromogenic media were not used for clinical specimens. Organism identification and susceptibility testing was performed using an automated commercial system (Vitek 2, bioMerieux Australia) with CLSI (Clinical and Laboratory Standards Institute, USA) breakpoints. To increase the sensitivity of detecting isolates with low levels of carbapenemase production, all clinical isolates were additionally screened with agar dilution methods with 0.5-1.0 mg/mL meropenem agar plate. Bacterial isolates which were resistant to meropenem on commercial testing or which grew on the agar dilution plate, and selected isolates that demonstrated cephalosporin resistance (e.g. ESBLs), were submitted for further testing with a Modified Hodge test and subsequent confirmatory testing utilizing an in-house developed multiplex molecular (PCR) testing for the presence of *bla-*IMP or *bla-*VIM [12].

Environmental samples were collected using swabs to sinks and drains. Engineering staff assisted with exposure to deep and enclosed sinks. Swabs were incubated directly into 10 mL of Brain Heart Infusion at the site of collection. Incubated broth was incubated at 37°C overnight and then plated onto chromogenic ESBL agars (Oxoid Brilliance Chrome ESBL) and incubated aerobically at 37°C for a further 24 hours [13]. Enterobacteriaceae isolated from this selective media underwent identification and susceptibility testing as well as meropenem agar dilution plate screening as described for clinical specimens above.

### Data collection

Laboratory data were collected from electronic databases. Clinical information was retrieved from medical records. BU data were obtained from the NSW Agency for Clinical Innovation Statewide Burns Injury Service.

### Statistical analysis

Bivariate and multivariate analyses were performed for age, total body surface area (TBSA) and length of stay, compared to likelihood of acquiring MBL organisms, using Stata 12 (StataCorp, Texas, USA).

## Results

### Clinical outbreak

**Figure 1:**
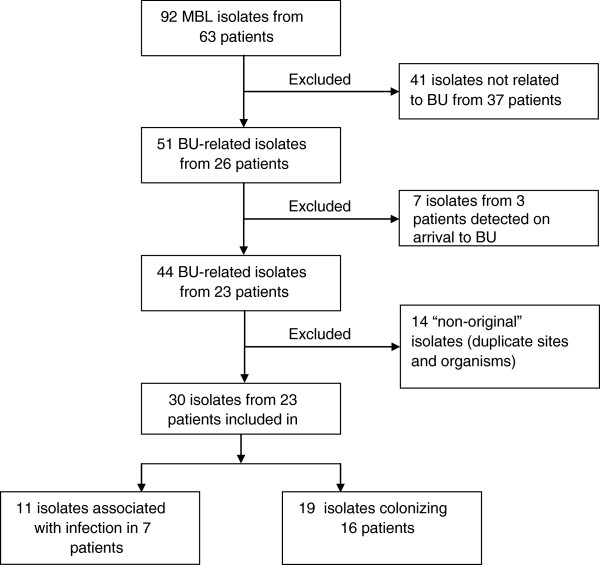
Flowchart of study selection criteria.

The CRGH Microbiology laboratory identified 92 MBL-producing isolates over the six-year period from 63 patients. All were *bla-*IMP-4 positive, except for one non-BU related VIM-1 isolate. Of these, 51 (55%) isolates from 26 patients had some temporal relationship to the BU. Thirty isolates (33%) from 23 patients met the case definition (Figure 1), representing 1.5% (23/1497) of BU patient admissions over this time.

All patients were admitted for burns management except for one, who had a diagnosis of necrotizing fasciitis. Eighteen (78%) of patients were male. Older age, larger total burns surface area (TBSA) and greater length of stay (LOS) were all significantly associated with MBL acquisition on both bivariate and multivariate analyses (p < 0.001), as summarized in Table 1. Median number of days in BU to acquisition of MBL organism was 25 days (range 7–87). Fourteen (61%) patients had an ICU admission prior to transfer into the BU, with a median ICU stay of 6 days. Eighteen (75%) patients were transferred from another hospital. Twenty-one (92%) patients had surgery during that admission, most commonly for debridement with or without split skin grafting.

**Table 1:** Risk factors of MBL acquisition in burns unit

**Risk factor**	**Bivariate analyses**			**Multivariate analysis**	
	**MBL**	**Controls**	***p***		
	***n*** **= 23**	***n*** **= 1,497**		**OR (95% CI)**	***p***
	**median (p50)**				
Age (years)	62	38	<0.001	1.04	<0.001
TBSA (%)	20	4	<0.001	1.03	<0.001
LOS (days)	50	6	<0.001	1.02	<0.001
LOS v TBSA	correlation coefficient (r) =0.741		<0.001		
LOS v age	correlation coefficient (r) =0.260		<0.001		
TBSA v age	correlation coefficient (r) =0.009		0.622		

*TBSA* Total Burns Surface Area (as percentage of total body surface area); *LOS* length of stay.

Fifteen (65%) patients had no known immunocompromise prior to admission. Three patients had a history of alcohol abuse, two had diabetes mellitus. One patient had polymyalgia rheumatica, one with lung cancer in remission and a previous cerebrovascular accident, and one patient had chronic obstructive pulmonary disease and peripheral vascular disease.

Sixteen (70%) patients had prior broad-spectrum antibiotics, defined as either third or fourth generation cephalosporins, and/or broad-spectrum penicillins such as ticarcillin-clavulanate or pipercillin-tazobactam, with a median duration of 7 days (range 1–80 days). Four of these patients also received carbapenems. Two patients received no antibiotics prior to MBL acquisition.

The majority of isolates (68%, n = 21) represented asymptomatic colonization. *Enterobacter cloacae* was the most commonly detected organism, seen in 61% (n = 19) of isolates in 16 patients. Other species identified were *Klebsiella pneumoniae* (8), *Enterobacter aerogenes* (2), and *Klebsiella oxytoca* (2). The most common sites of isolation were superficial wound swabs from the burns site (71%, n = 22), others included central venous catheter tips (4), peripheral blood (2), urine (2) and respiratory (1) cultures.

Table 2 summarizes the characteristics, treatment and outcomes of the 7 (30%) patients with clinical infections attributable to MBL organisms. Amikacin was given in all 6 patients who remained in the hospital, 3 of those in combination with ciprofloxacin.

**Table 2:** Characteristics and outcomes of 7 patients with clinically significant infections

**Patient No.**	**Age and Sex**	**Co-morbidities**	**Isolate site (n = 11)**	**Organism**	**Treatment**	**30-day Mortality**
1	62 M	Nil	CVC tip	*Klebsiella pneumoniae*	Amikacin 4 days	Survived
2	61 M	Alcohol abuse	CVC tip	*Enterobacter cloacae*	Amikacin 14 days	Survived
			Blood Sputum		Ciprofloxacin 14 days	
3	36 M	Alcohol abuse	Wound	*Enterobacter cloacae*	Surgical wound debridement, with 1 dose amikacin at time of operation	Survived
4	82 F	COPD, PVD	CVC tip	*Enterobacter cloacae*	Amikacin 5 days	Survived*
5	62 M	Nil	Wound	*Enterobacter cloacae*	Amikacin 8 days, ciprofloxacin 7 days	Survived
			Wound	*Klebsiella pneumoniae*		
6	86 F	Nil	Wound	*Enterobacter cloacae*	Stat dose amikacin, ciprofloxacin 6 days	Survived
7	92 M	Nil known	Blood	*Klebsiella pneumoniae*	Unknown	Unknown
			Urine			

* = died at 34 days after MBL infection, from respiratory failure related to inhalation injury, not attributed to MBL infection.

Of the known outcomes, 5 patients survived and were discharged. The single patient who died had MBL *E. cloacae* identified from CVC tip cultures. She was treated with amikacin for 5 days, subsequent blood cultures were positive for methicillin-resistant Staphylococcus aureus (MRSA) but no Gram-negative organisms. She died one month after treatment for MBL infection from respiratory failure, in the context of inhalational injury and underlying chronic obstructive pulmonary disease. Her cause of death was not deemed attributable to MBL infection. Patient 7 had positive blood cultures detected on the day he was transferred overseas for further care; it is documented that the result of an MBL-producing *Klebsiella pneumoniae* was communicated to the overseas hospital, however his treatment and outcome are unknown.

### Environmental screening

**Figure 2:**
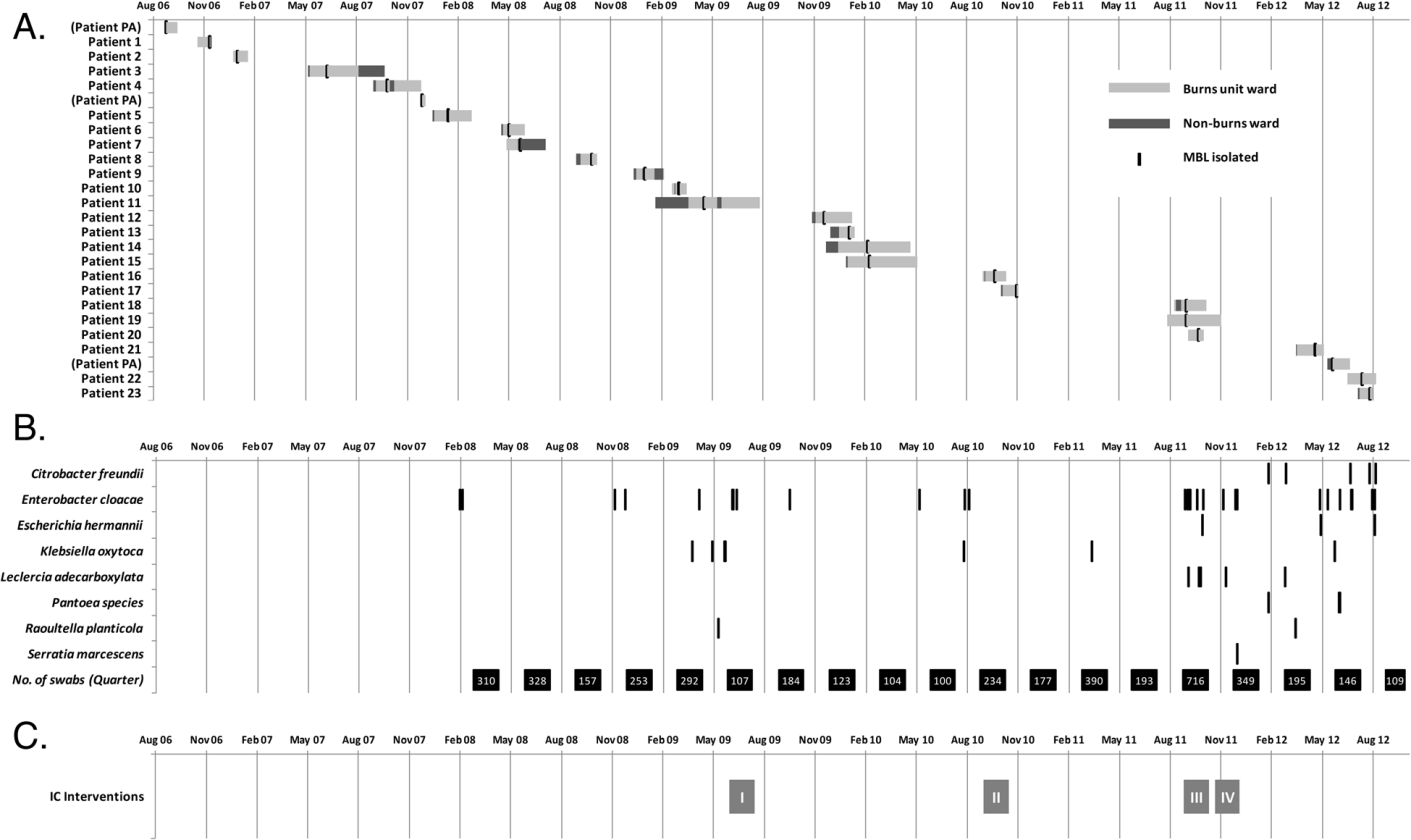
**Temporal relationship during the prolonged outbreak comparing A) clinical, B) environmental and C) infection control interventions; section B includes overall number of swabs taken during each quarter.** Infection control (IC) interventions were; I. Access of deep drains for cleaning; II. Extensive cleaning of all sinks and drains; III. Introduction of regular environmental surveys; IV. Terminal cleaning of the Burns Unit; PA = denotes patients with known MBL colonization prior to transfer into Burns Unit.

Environmental screening for MROs conducted in the BU has historically been done on an intermittent basis, across a range of randomly selected environmental sites within the BU. Sampling was usually performed from drainage areas and shower heads in common shower areas, and in rooms of patients known to harbor MROs following cleaning with a local disinfectant and a phenolic compound. In September 2011, a point-prevalence study was performed within the unit after a cluster of three clinical cases was detected over a short period of time. This additional sampling accounted for a significantly larger amount of overall swabs taken, and thus of positive MBL isolates (see Figure 2). In response to this finding, monthly routine screening was instituted. Defined sampling of environmental surfaces included patient rooms, shared patient equipment such as hoist chairs, bathroom facilities and plumbing, including shower floor drains and en-suite bathroom sink drains. Staff areas were also sampled. This was downgraded to bi-monthly screening from January 2012, and has continued until the time of writing.

**Figure 3:**
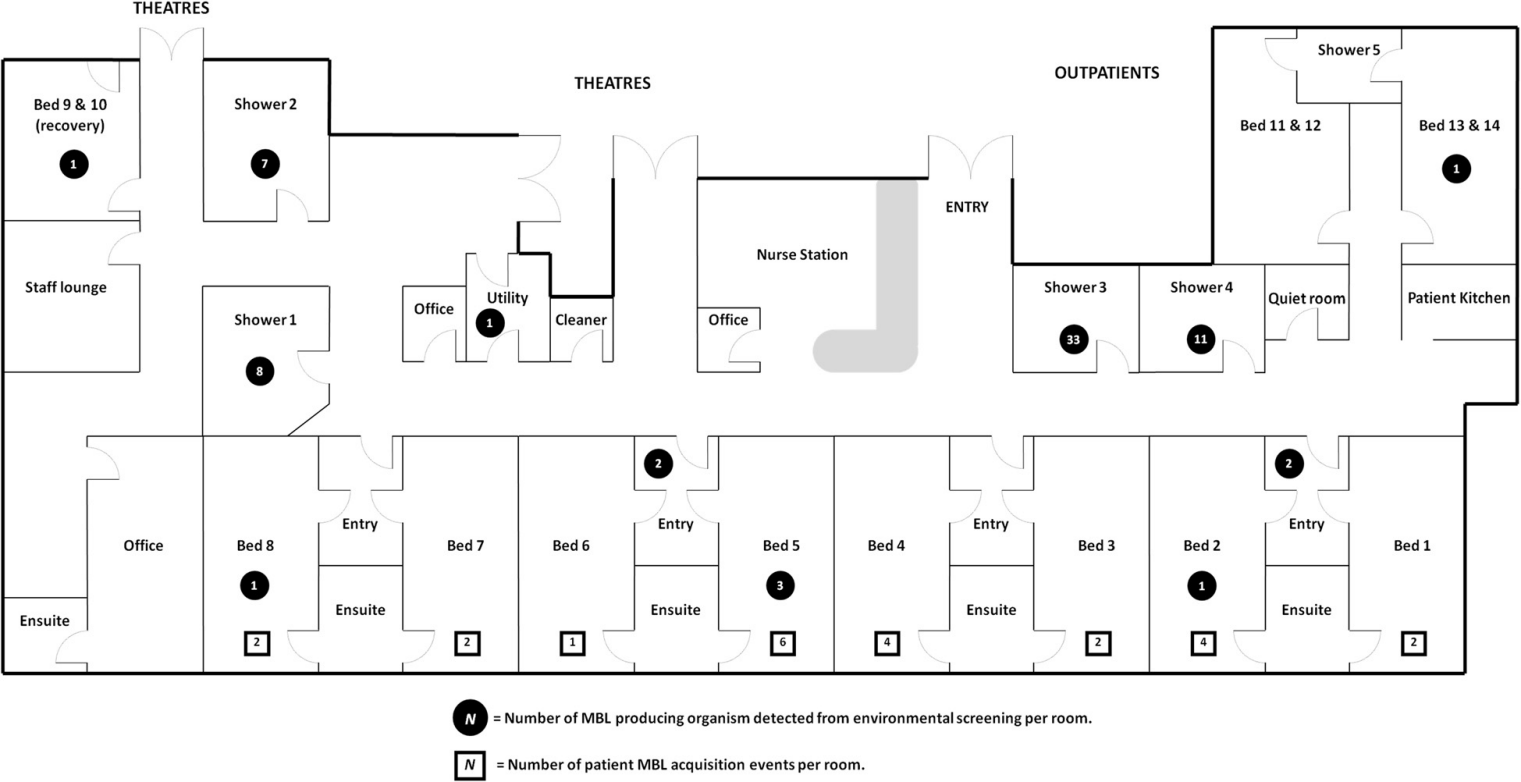
Map of BU plotting MBL environmental isolates, compared against rooms dwelled by MBL positive patients.

Table 3 summarizes the confirmed MBL isolates detected from environmental screening of the BU over this period. Since 2008 when molecular confirmation for MBL with IMP-4 PCR testing was instituted on environmental isolates, 4495 isolates have been collected, with 71 MBL positive isolates detected. *Enterobacter cloacae* was the predominant organism (56%, n = 40), in a similar proportion to the clinical isolates. Shower plumbing and equipment represented 85% (n = 60) of environmental isolates (Table 4). Despite sampling, MBL isolates were not identified in any area not directly related to patient contact or water drainage, e.g. nurses’ workstation, staff room or office areas. Figure 3 describes the close spatial relationship between sites where environmental MBL isolates were detected, especially the shower facilities, compared to the patient rooms in which the study patients had dwelled at the time of MBL acquisition. Figure 2 describes the temporal relationship between MBL positive patients admitted into the BU, against environmental isolates detected over the same time period. It also denotes the instances when additional intensive hospital cleaning measures were implemented, in an attempt to eradicate MBL colonization within the unit.

**Table 3:** **Clinical and environmental *bla*-IMP-4 MBL isolates by species**

**Species**	**Clinical Isolates (n = 30)**	**Environmental Isolates (n = 71)**
*Enterobacter cloacae*	19	40
*Enterobacter aerogenes*	1	0
*Klebsiella oxytoca*	2	8
*Klebsiella pneumoniae*	8	0
*Leclercia adecarboxylata*	0	7
*Citrobacter freundii*	0	6
*Escherichia hermannii*	0	3
*Pantoea species*	0	4
*Raoultella planticola*	0	2
*Serratia marcescens*	0	1

**Table 4:** **Environmental *bla*-IMP-4 MBL isolates by site type**

**By Site Type**	**N = 71**
Shower Plumbing (e.g. drains, taps, sinks)	38
Shower Equipment (e.g. trolley, chair, curtain)	22
Room Plumbing (e.g. handwashing sink, taps)	4
Room Equipment (e.g. bed rail, suction equipment)	6
Other	1

### Infection control interventions

Single room isolation and standard contact precautions (with donning gowns and gloves in an anteroom prior to entry) were pre-existing prior to introduction of MBL organisms into the BU. There was good hand hygiene compliance amongst staff, and the hospital had an established antibiotic stewardship and approval program. Interventions in response to the outbreak have therefore focused on hospital cleaning and engineering solutions, especially as MBL organisms were primarily isolated in recurring environmental niches.

In response to a growing number of MBL organisms being found from drain sites, liaison with engineering services allowed access by cleaning staff into floor drainage and sink traps that were previously screwed down, and these deep drains were terminally cleaned by hospital plumbers. In September 2010, regular physical cleaning of drains to remove biofilm and additional cleaning with double-strength phenolic disinfectant Phensol™ was adopted. This was changed to the chlorine-based product Chlor-clean™ in April 2012. In September 2011, point-prevalence surveillance of the environment was performed to access the situation, in consultation with the burns department, infection control, environmental and engineering services. After this survey revealed ongoing contamination of the unit, a concerted effort for terminal cleaning of the Unit was performed in October 2011, and implementation of monthly routine surveillance, including sampling both before and after hospital cleaning. Despite this, 12 (32%) of the 37 positive environmental isolates since October 2011 were samples obtained after cleaning.

## Discussion

We describe the persistence of MBL-producing organisms in a nosocomial setting, detected from both clinical and environmental sources, which has continued for six years. A small but significant proportion of patients admitted to BU were colonized and/or infected, 1.5%, and BU-related isolates were greatly overrepresented, accounting for 55% of overall hospital MBL isolates detected during this time. Case patients were significantly older, with greater extent of burns and hospital lengths of stay. Those with clinical infections had generally favorable outcomes, with no attributable deaths. Most cases reflected asymptomatic colonization rather than clinical infection, in contrast to a previously described outbreak in the Intensive Care Unit at another Australian hospital, where 75% of patients carrying MBL-organisms had attributable infections [5].

Between 2000 and 2006, the predominant carbapenem-resistant Gram-negative organism isolated from BU environmental screening was multidrug-resistant *Acinetobacter baumannii*, conferring resistance via a Class D OXA-23 enzyme. Its presence was curtailed with improved environmental sampling methods [13] together with an effective surface cleaning intervention, which included hiring of dedicated cleaners for the BU. Consequently MDR *A. baumannii* ceased to be detected from the environment after this time.

Environmentally-isolated CRE were first detected in our BU in January 2007, however molecular IMP-4 MBL confirmation in environmental isolates had not been instituted at that time, and these unconfirmed carbapenem-resistant isolates were excluded in this study. Since molecular confirmation began in early 2008, MBL organisms have been recurrently isolated, predominately within the shower facilities that are shared amongst the patients. Our findings of multi-resistant Gram-negative organisms persisting in mostly wet areas despite hospital disinfection, echoes the experiences described both locally [7], and overseas [8,9,14,15], of outbreaks related to plumbing and water-borne sources [16]. These studies, as well as genomic data from selected clinical and environmental isolates from our BU [11], suggest a close association between environmental sources, colonization and secondary infection. However, unlike outbreaks that have largely centered around handwashing sinks, the vast majority of positive environmental isolates originated from drains and sluices within the BU shower facilities, though not the shower heads themselves. We postulate that severe burns patients’ large surface areas of denuded and exposed skin, and requirement for regular bathing, puts them at greater risk of colonization by environmental Gram-negative organisms that may have a water-related reservoir [17], particularly if associated with biofilms [8].

While some studies have found implementation of infection control bundled interventions, such as universal standard precautions with antibiotic stewardship, have eradicated local outbreaks of multi-resistant Gram-negative organisms [6,18], others have experienced outbreaks despite this [19]. Existence of such measures in our BU well before outbreak onset did not prevent its establishment or dissemination, although it may have limited its potential magnitude. Although terminal cleaning within our BU terminated an MDR-*Acinetobacter baumannii* outbreak six years previously, repeated attempts at cleaning interventions targeting structural plumbing components have not resulted in elimination of MBLs from the environment. This may be related to the persistence of biofilms despite terminal cleaning [20]. CRE outbreaks in other settings have involved physical removal of plumbing, together with other infection control measures, with subsequent success at terminating the outbreak [9,15].

We recognize that the association of MBLs detected in clinical and environmental isolates cannot prove causality. In fact this has, until recently, hampered discussion of the role of the environment in transmission of Enterobacteriaceae between patients and the importance of hospital cleaning in control. Even though the MBL isolates in both clinical and environmental samples were shown to be genetically identical [11], this does not confirm the reservoir as the definitive source of transmission to newly admitted patients. Nonetheless, apart from the BU staff, the only environment shared between patients, were the shower and bathroom facilities. There was continuing environmental detection and despite concerted terminal cleaning, persistence of MBLs in these, independent of the concomitant presence in the BU of MBL positive patients. We believe the consistency of our findings in a unique patient niche, in association with similar findings emerging in the literature to echo our experience [9,15] would suggest that environmental reservoirs for multi-resistant Enterobacteriaceae are a potential source for ongoing cross-contamination.

Novel approaches to environmental disinfection, such as hydrogen peroxide vapor decontamination [21,22] are being trialed, however this approach may be more efficacious against surface environmental organisms such as MRSA and VRE, and efficacy against Enterobacteriaceae, especially those with an established water source, have not been demonstrated to date.

One limitation to this retrospective study was the irregular nature of surveillance from both clinical and environmental sources, which potentially missed a proportion of asymptomatic colonization, and potential environmental reservoirs, due to the selective nature of environmental sampling. This study highlights the importance for active surveillance during an outbreak and in high-risk settings such as the Burns Unit, as recommended by the Centre of Disease Control regarding the control of CREs [23]. A recent carbapenem-resistant *Klebsiella pneumoniae* outbreak at a National Institutes of Health hospital involved whole-genome sequencing to track the origin of the outbreak and delineate its epidemiology [10]. Real-time sequencing may prove a useful tool in the future as cost-effectiveness and technological improvements may lead to mainstream clinical use.

Laboratory detection methods carried out at the beginning of the outbreak may have under-detected the burden of MBLs in both clinical and environmental isolates, as automated systems may miss carbapenemase-producing organisms [24,25], and CLSI breakpoints were lowered in 2010 to facilitate greater detection of CREs [26]. Despite this, we observed that improving the sensitivity of laboratory CRE detection, particularly for environmental CRE organisms, required the addition of a lower MIC (0.5 mg/L meropenem) agar dilution plate. Developing rapid diagnostic tests to detect CREs is difficult, especially for environmental screening purposes, due to their multiple and complex resistance mechanisms [27], and also in screening a multitude of organisms, some of which may only exhibit low-level resistance. Such a test would allow prompt identification of carrier patients, incur appropriate infection control measures, and delineate the extent outbreak before its spread. We have recently evaluated the CarbaNP test as a simple, cost-effective test for CRE testing [28] and hope to implement this in routine environmental screening for *bla*-IMP-4 organisms.

Further studies are required to explore the long-term outcome of patients colonized with MBL organisms, and whether chronic carriage persists after hospital discharge. The persistence of CRE in the nosocomial setting despite traditional measures signal a need to pursue newer approaches, including novel cleaning methods, assessing the need for removing physical structures, such as plumbing and drains, vulnerable to biofilm formation, and the role active surveillance plays in monitoring and curtailing outbreaks.

## Competing interests

The authors declare that they have no competing interest.

## Authors’ contribution

GL, EC and TG conceived the study design. GL performed the data collection and analysis, and drafted the manuscript. TJG contributed to detailing the microbiological methods in the manuscript, and drafted the figures in this manuscript. EC, TG and TJG revised drafts of the manuscript. All authors read and approved the final manuscript.
